# Supplementary material for: An acidophilic fungus promotes prey digestion in a carnivorous plant
Source: Nat Microbiol. 2024 Aug 1;9(10):2522–37. doi: 10.1038/s41564-024-01766-y (PMC11445062; doi:10.1038/s41564-024-01766-y)

nature microbiology

Supplementary information https://doi.org/10.1038/s41564-024-01766-y

An acidophilic fungus promotes prey digestion in a carnivorous plant

In the format provided by the authors and unedited

An acidophilic fungus promotes prey digestion in a carnivorous plant

Pei-Feng Sun, Min R Lu, Yu-Ching Liu, Brandon J. P. Shaw, Chieh-Ping Lin, Hung-Wei Chen, Yu-fei Lin, Daphne Z. Hoh, Huei-Mien Ke, I-Fan Wang, Mei-Yeh Jade Lu, Erica B. Young, Jonathan Millett, Roland Kirschner, Ying-Chung Jimmy Lin, Ying-Lan Chen and Isheng Jason Tsai

Correspondence: Isheng Jason Tsai ijtsai@gate.sinica.edu.tw

Supplementary Figures

Supplementary Fig. 1. *Drosera spatulata* in its natural habitat. a. *D. spatulata* typically grows on the cliff habitat of northern Taiwan. **b.** Close-up of *D. spatulata* growing surrounded by grasses and moss.

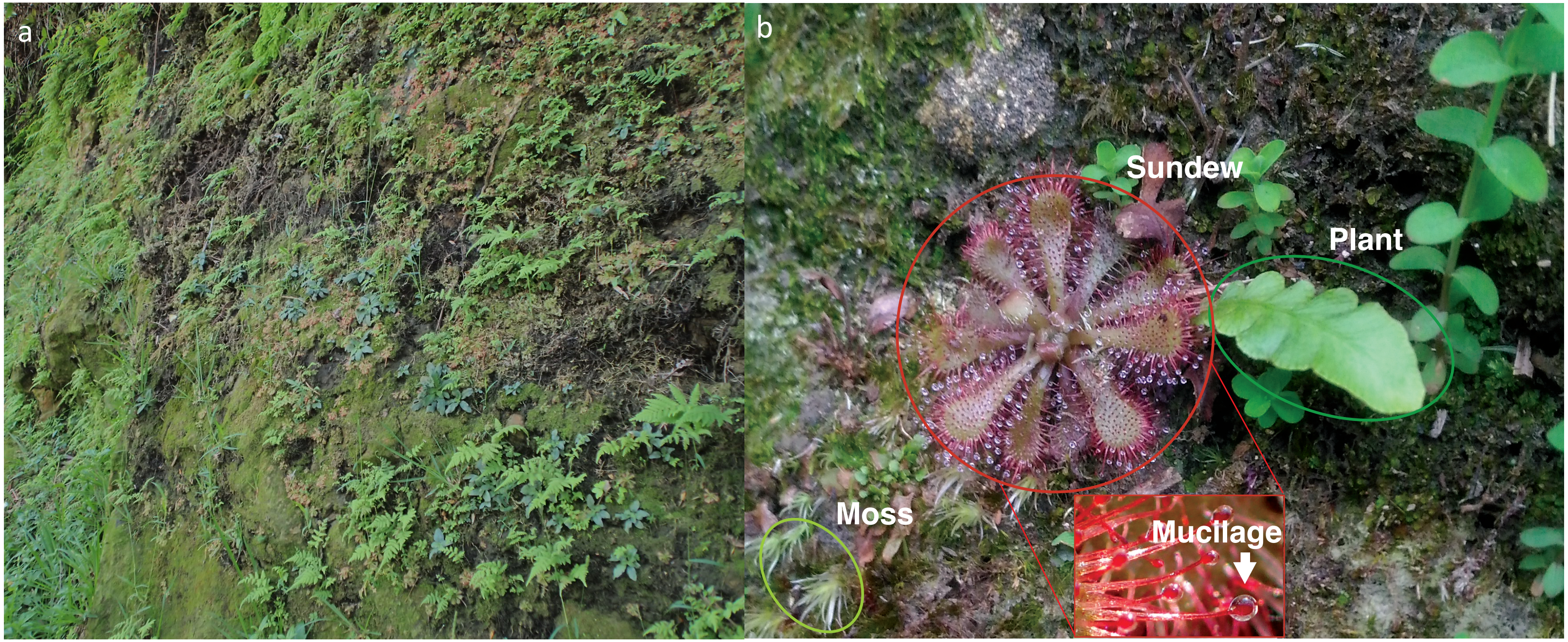

Supplementary Fig. 2. Sampling locations of initial *D. spatulata* mucilage survey. Numbers in bracket denote samples collected in each location. Each dot is a different region separated by at least 3.5 km.

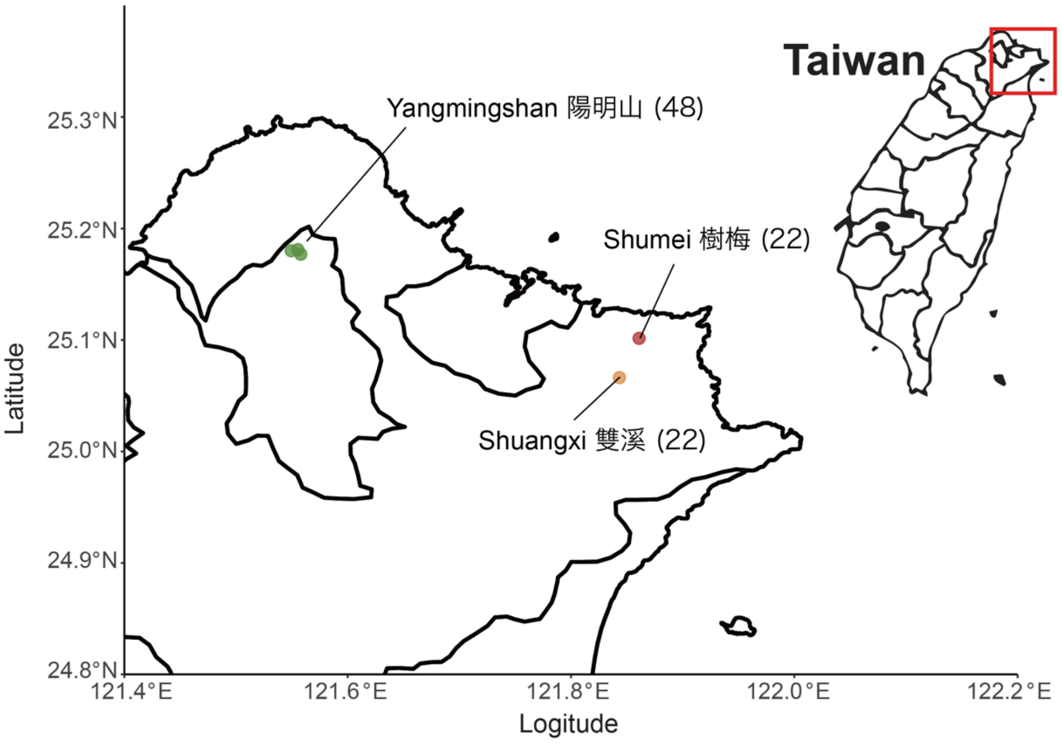

Supplementary Fig. 3. The growth profile of *A. crateriforme* and *P. herbarum* in different a. pH values (n=12 plates) and b. temperatures (n=40 plates). c. Temperature data in Shumei and Shuangxi from Taiwan Central Weather Administration. d. Weight of *D. spatulata* plants (n=10) one month after inoculated with different fungi. The centre line of the boxplot represents the median, with the upper and lower borders of the box marking the 25th and 75th percentiles, respectively. Whiskers extend to 1.5 × i.q.r.

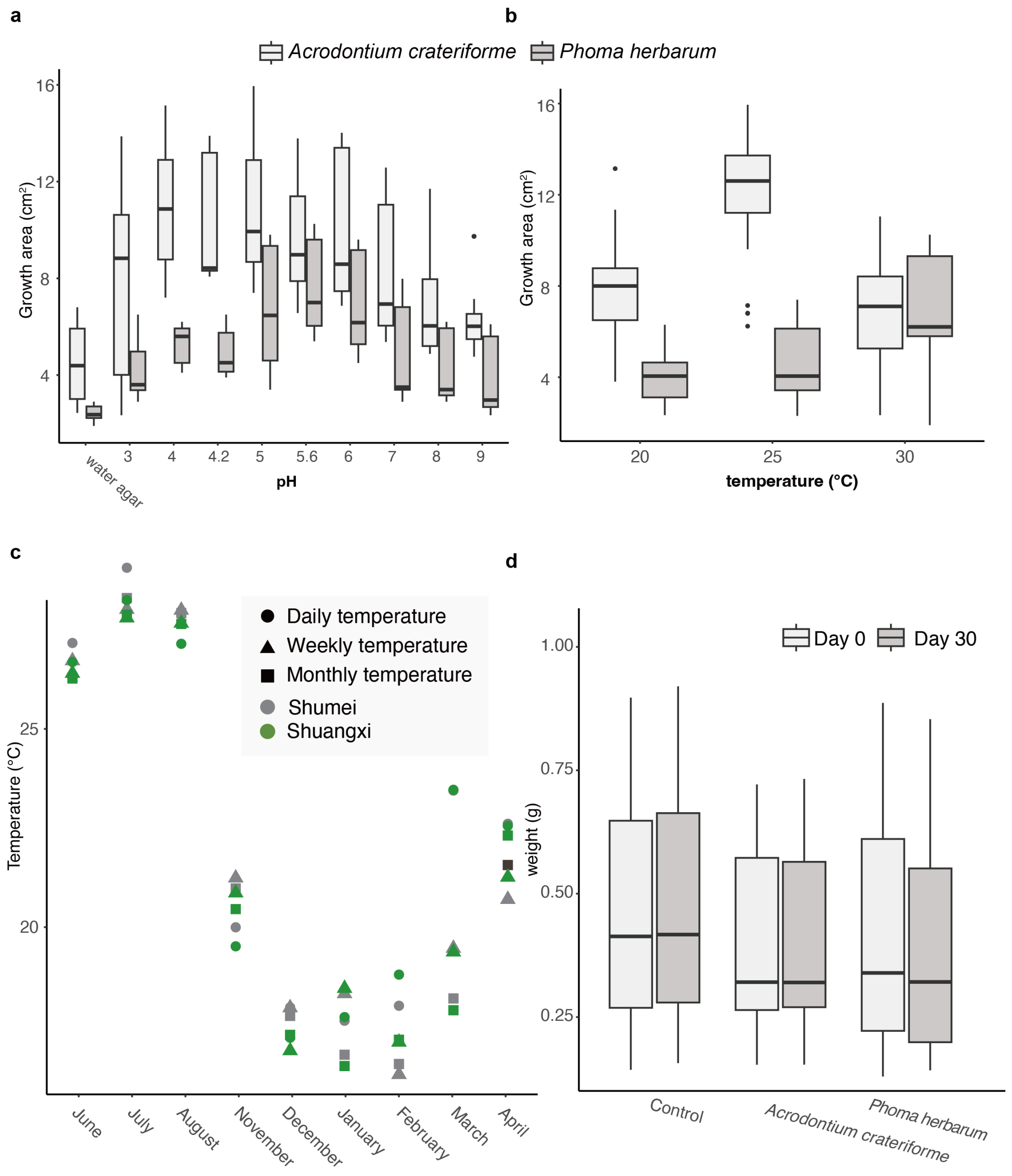

Supplementary Fig. 4. Growth profiling of *A. crateriforme* in 1/2 MS media with and without supplementing ant powder (n=6 plates). Data are presented as mean values +/- SD.

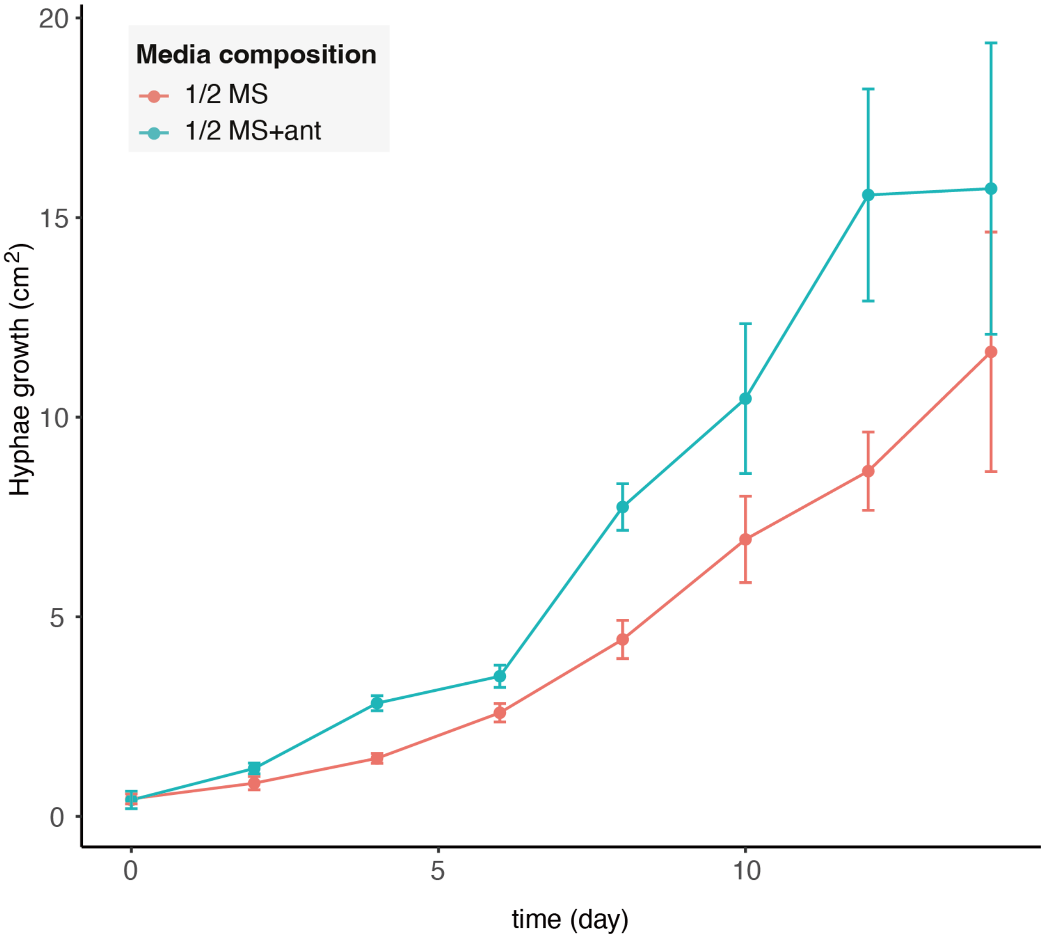

Supplementary Fig. 5. Western blotting image of biotin-BSA digestion in mucilage under different treatment. Raw images of subsequent blotting are shown in **Supplementary Fig. 22-24 which** were used to calculate the values used in **Fig. 3e** and **Extended Fig. 6**.
Unstained protein ladder: Thermo 26630, Prestained protein ladder: Thermo 26616. Negative control indicated pH4 MES buffer. Positive control indicated biotin-BSA in pH7 or pH4 of MES buffer. *Drosera* mucilage + biotin-BSA shows *Drosera* mucilage mixed with biotin-BSA in pH4 MES buffer. *Drosera* mucilage shows *Drosera* mucilage in pH4 of MES buffer.

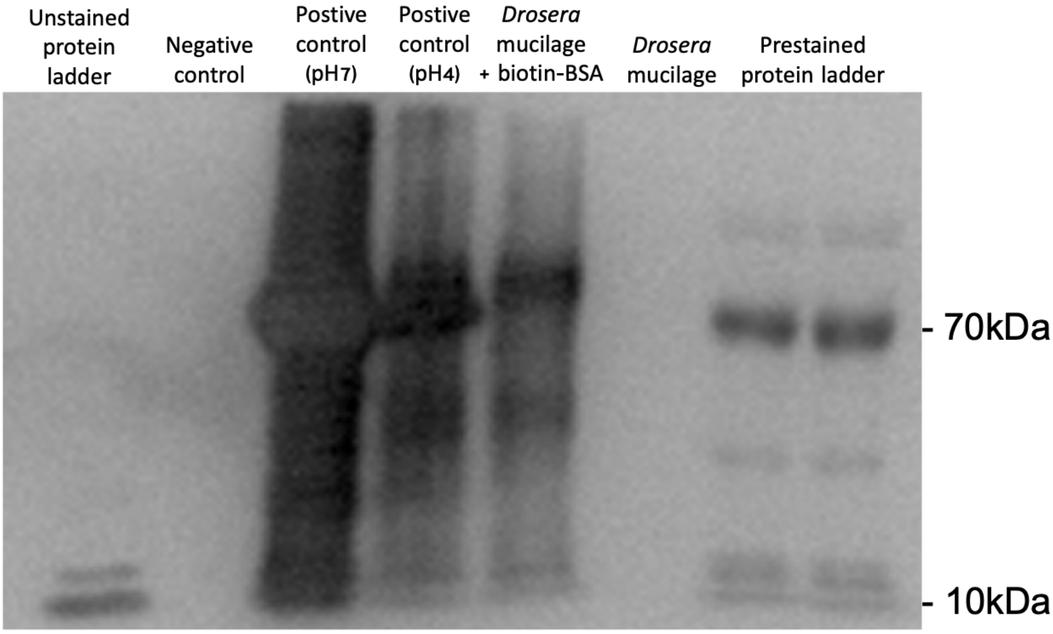

Supplementary Fig. 6. The gene distributions around mating type (MAT) related genes.

The mating locus and related orthologs were identified by BLAST the mating related gene sequences retrieved from *Aylward* et al against the *A. crateriforme* gene predictions. Within the *A. crateriforme* assembly, were located adjacent to each other and inferred as putative mating locus. We identified the orthologs of MAT genes and related APC5 and APN2 genes which are adjacent to each other and displayed in synteny with sister species. This region was inferred as putative mating locus and the gene distributions around MAT, APC5, and APN2 genes (including 5kb up- and down-stream flanking regions) are drawn.

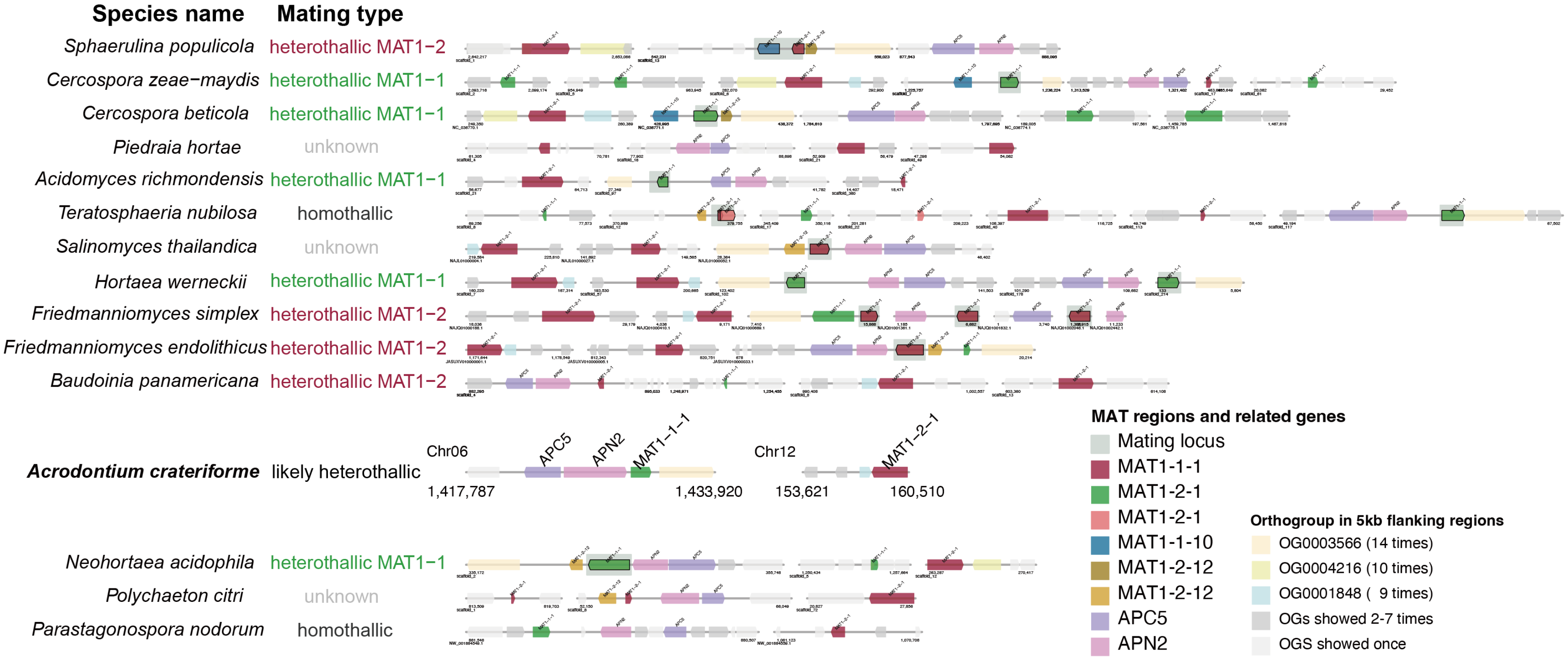

Supplementary Fig. 7. PCA of protein family domain numbers from 25 fungal species a. PCA of all species, **b.** zoomed in plot. Species full names corresponding to abbreviations are available in **Supplementary Table 4**

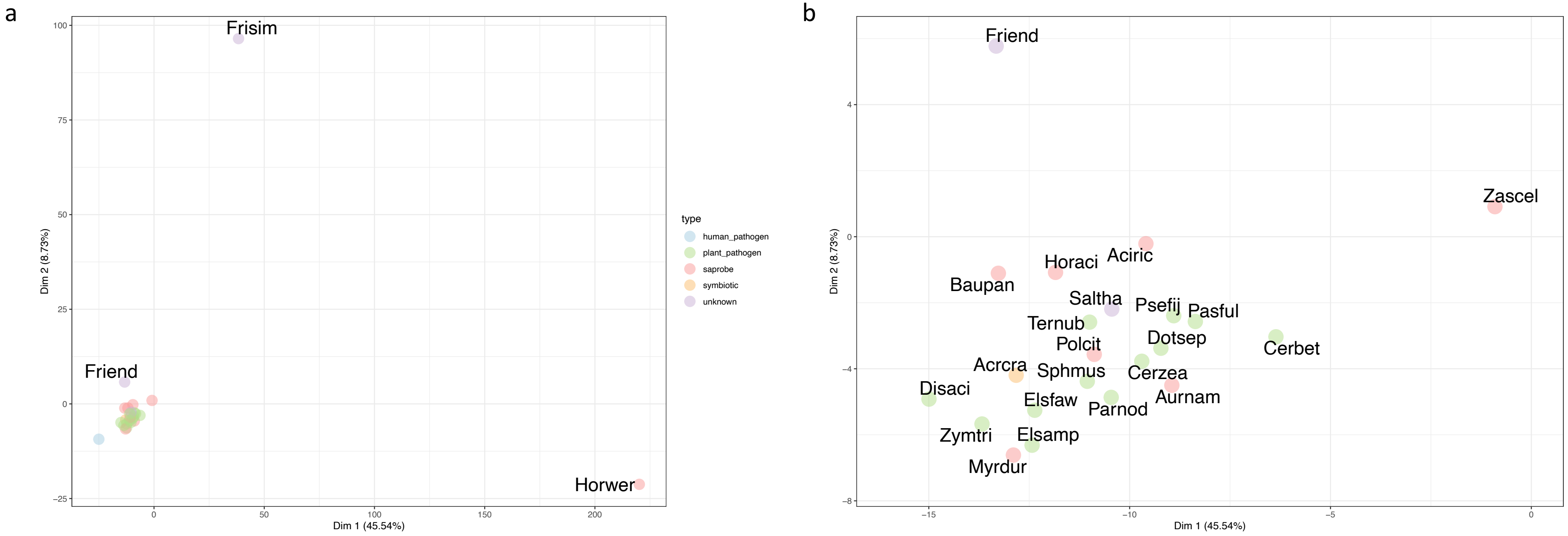

Supplementary Figure 8. Heatmap of biosynthetic gene clusters (BGC) in 25 fungal species.

Figure only showed regions with more than 50% orthology to at least one another region.

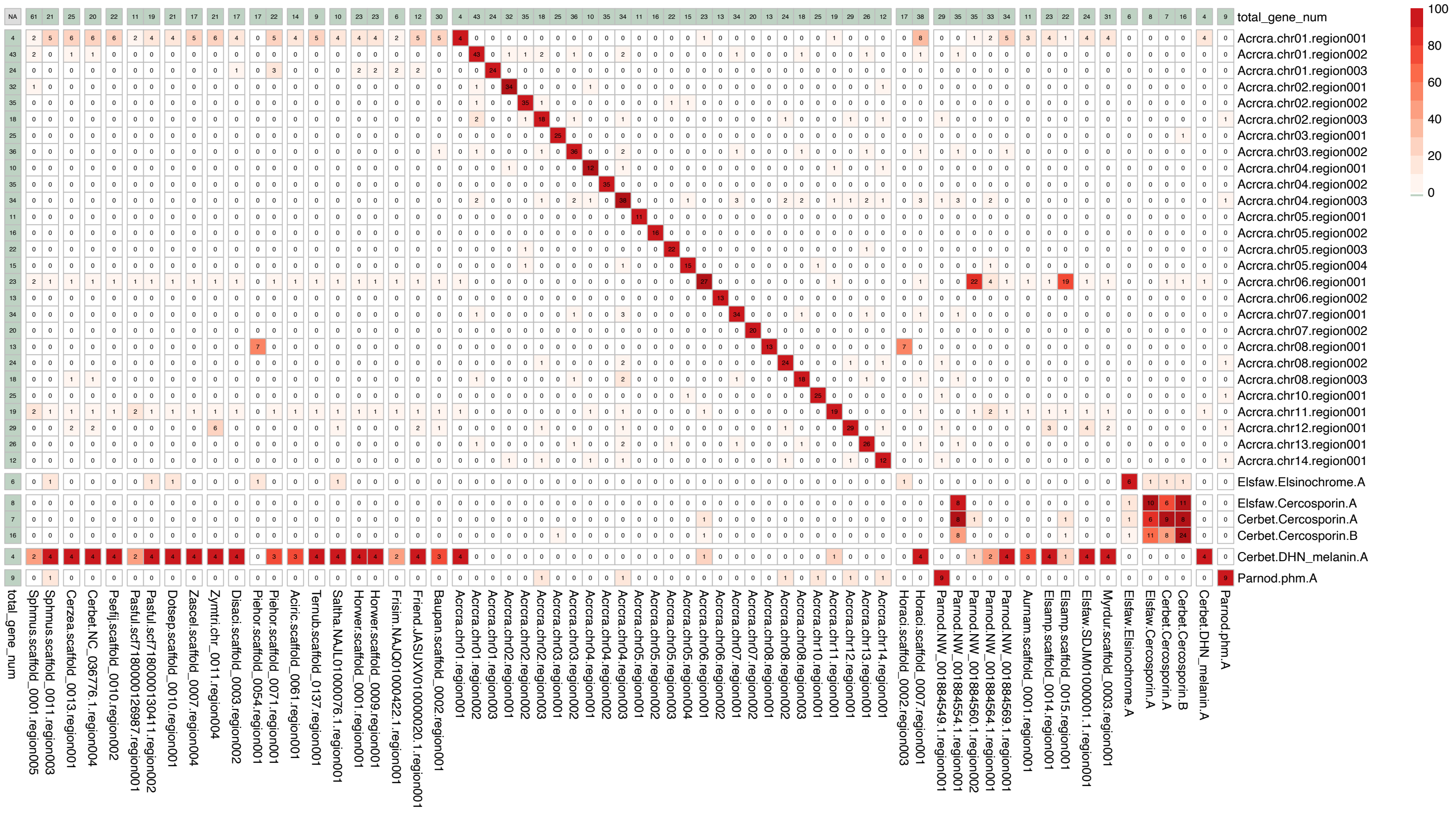

Supplementary Fig. 9. Top 20 Pfam gain and loss of *A. crateriforme*.

Gains and losses were ranked by domain frequencies. A z-score was calculated for the corresponding abundance of every domain in each species. Species full names corresponding to abbreviations are available in **Supplementary Table 5**.

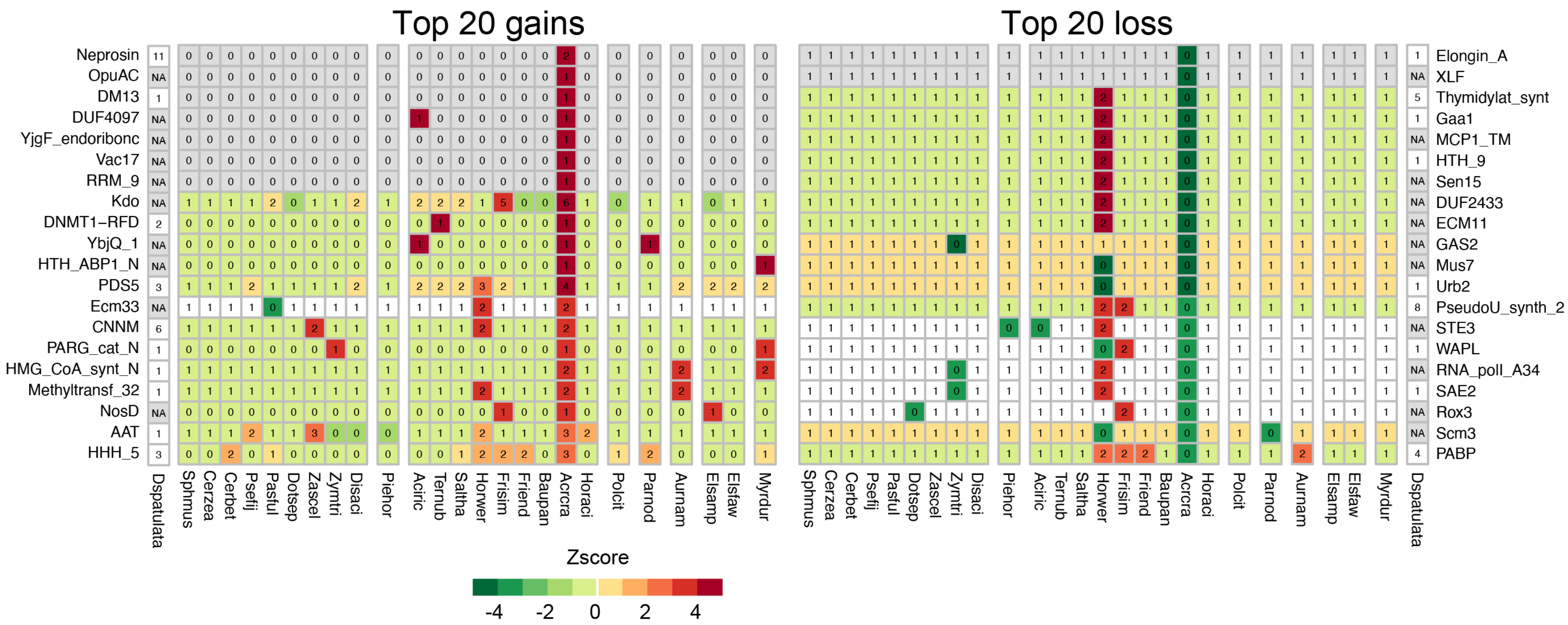

Supplementary Fig. 10. Visualisation of gene order within linkage groups has been lost between *A. crateriforme* and sister species. Synteny blocks were determined with DAGchainer (ver. r120920) and visualized via CIRCOS (ver. 0.69.9).

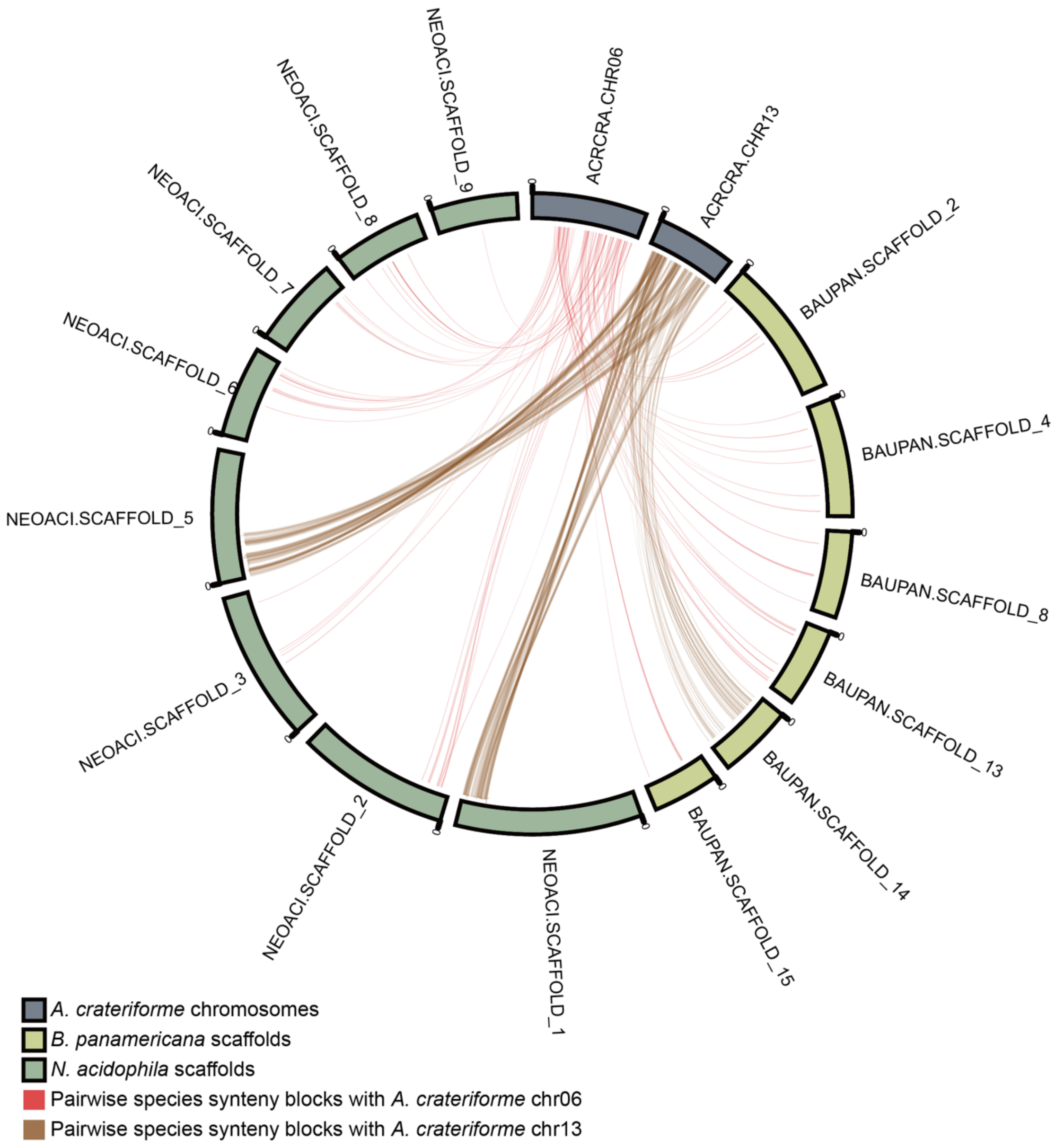

Supplementary Fig. 11. Overlap of top 20 enriched GO terms in *A. crateriforme* and *D. spatulata* under different conditions.

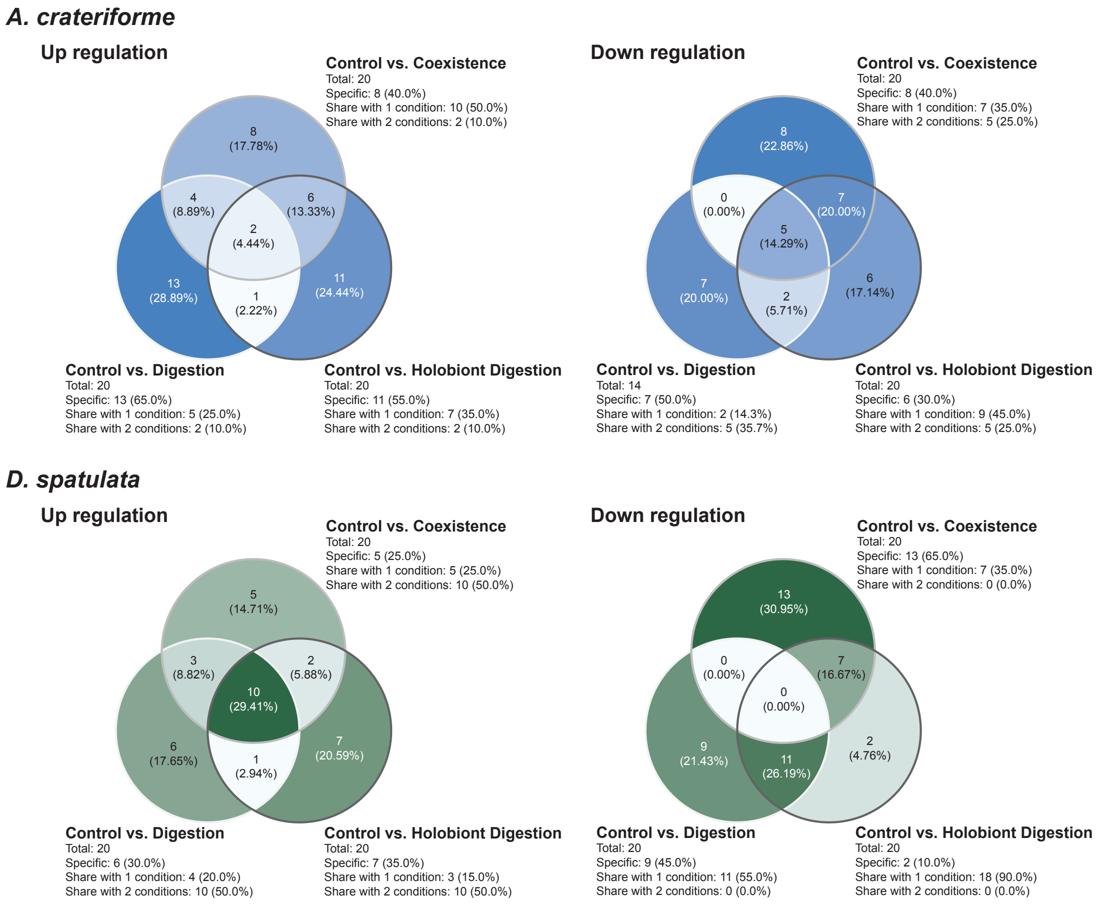

Supplementary Fig. 12. Expression of sundew chitinase in different treatments

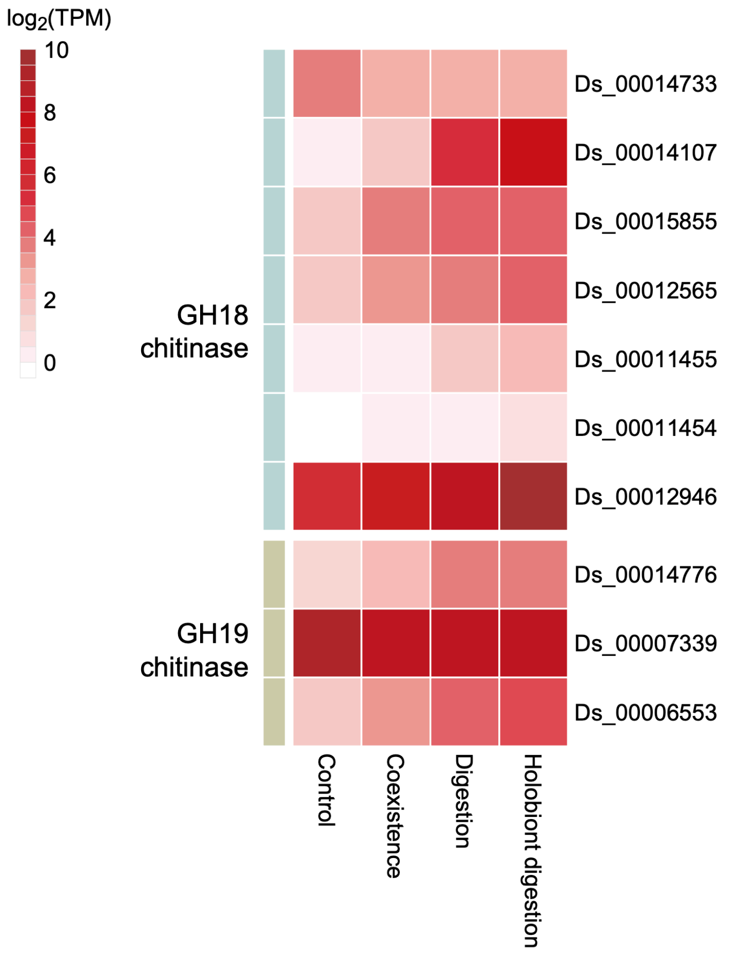

Supplementary Fig. 13. Expression of ammonium transporters in different treatments

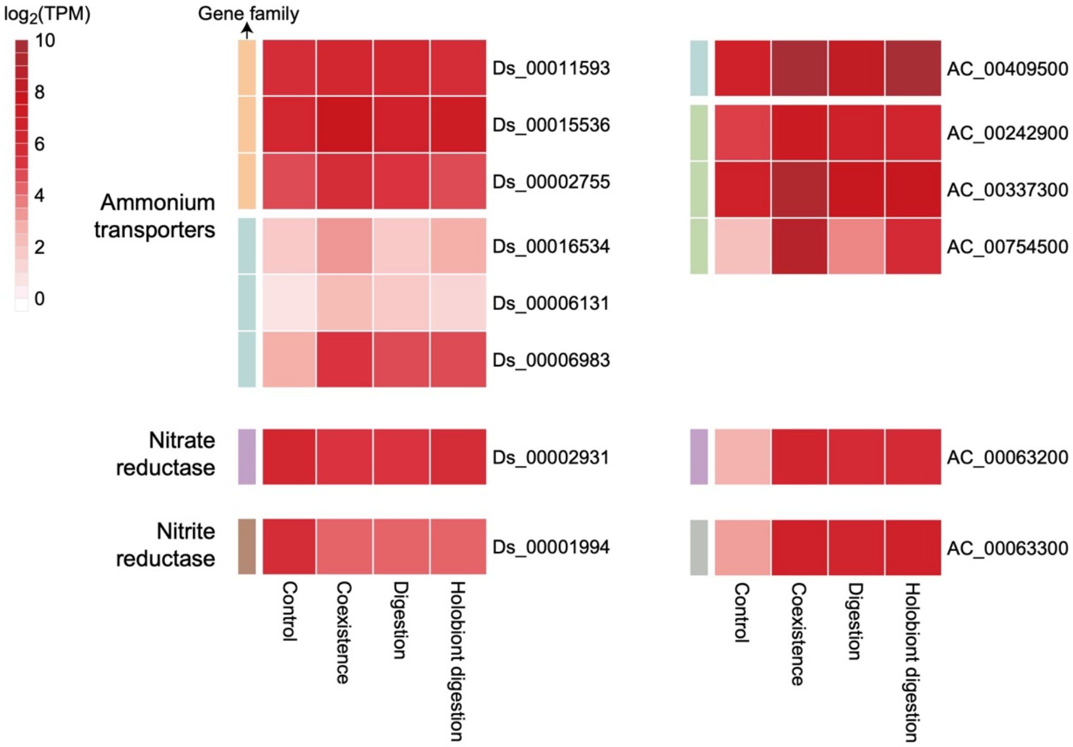

Supplementary Fig. 14. Upregulation of a BGC on chromosome six in *A. crateriforme*.

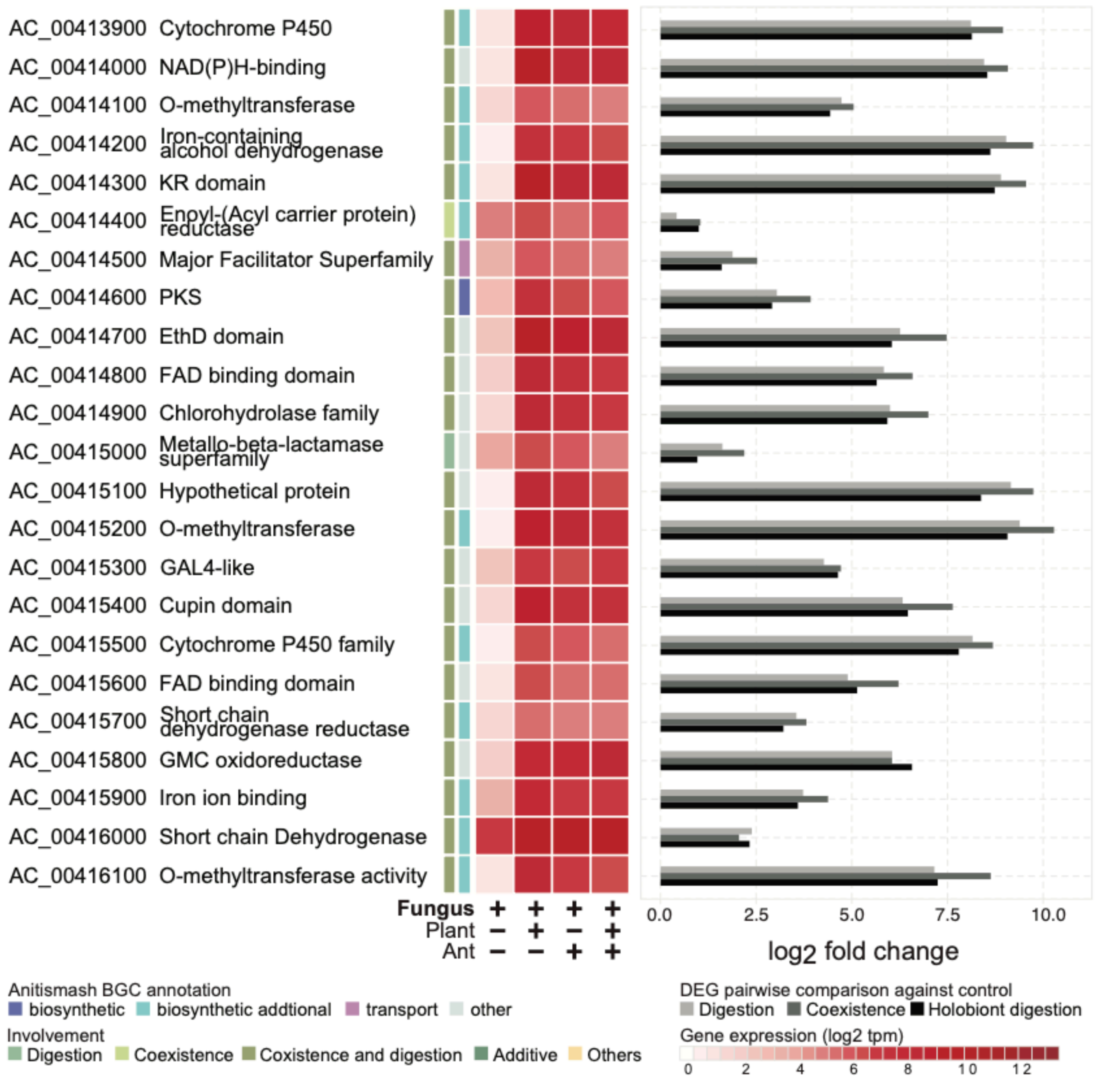

Supplementary Fig. 15. Expression of asparagine synthetase in different treatments

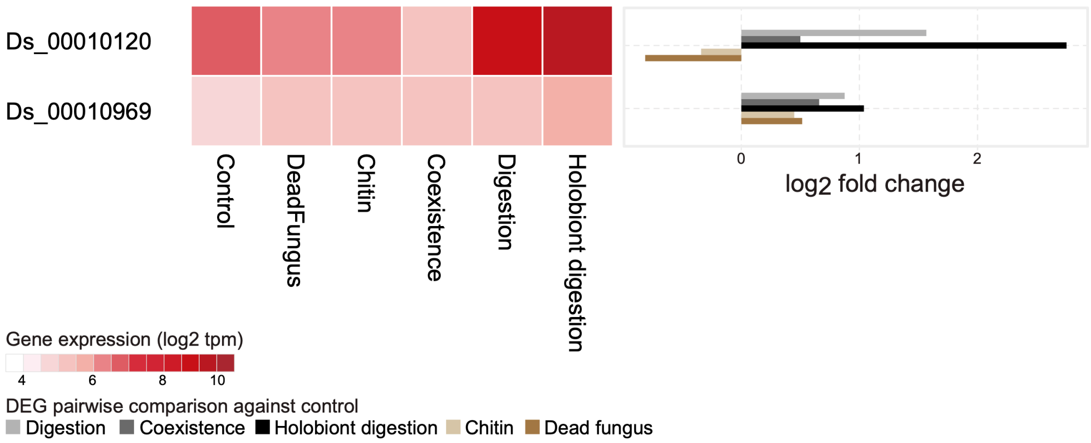

Supplementary Fig. 16. Co-expression gene modules in *A. crateriforme* across digestion and coexistence conditions using the weighted correlation network analysis (WGCNA)

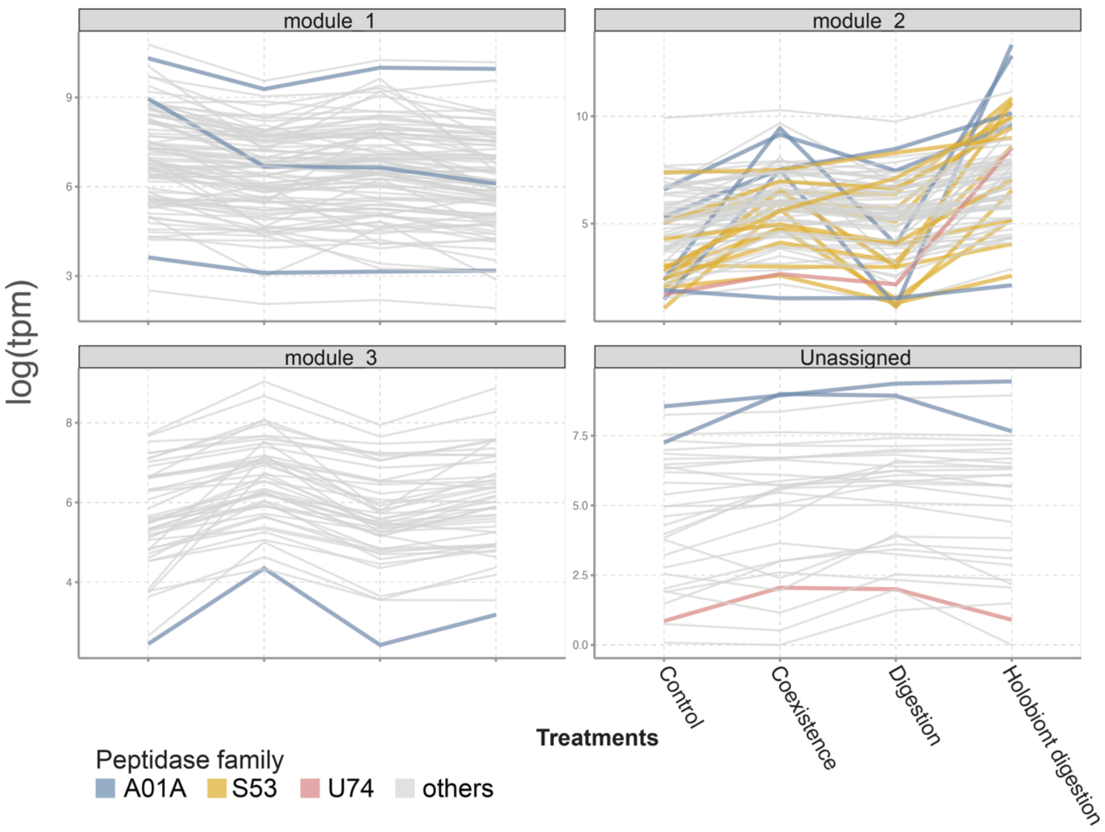

Supplementary Fig. 17. Co-expression gene modules in *D. spatulata* across digestion and coexistence conditions using the weighted correlation network analysis (WGCNA)

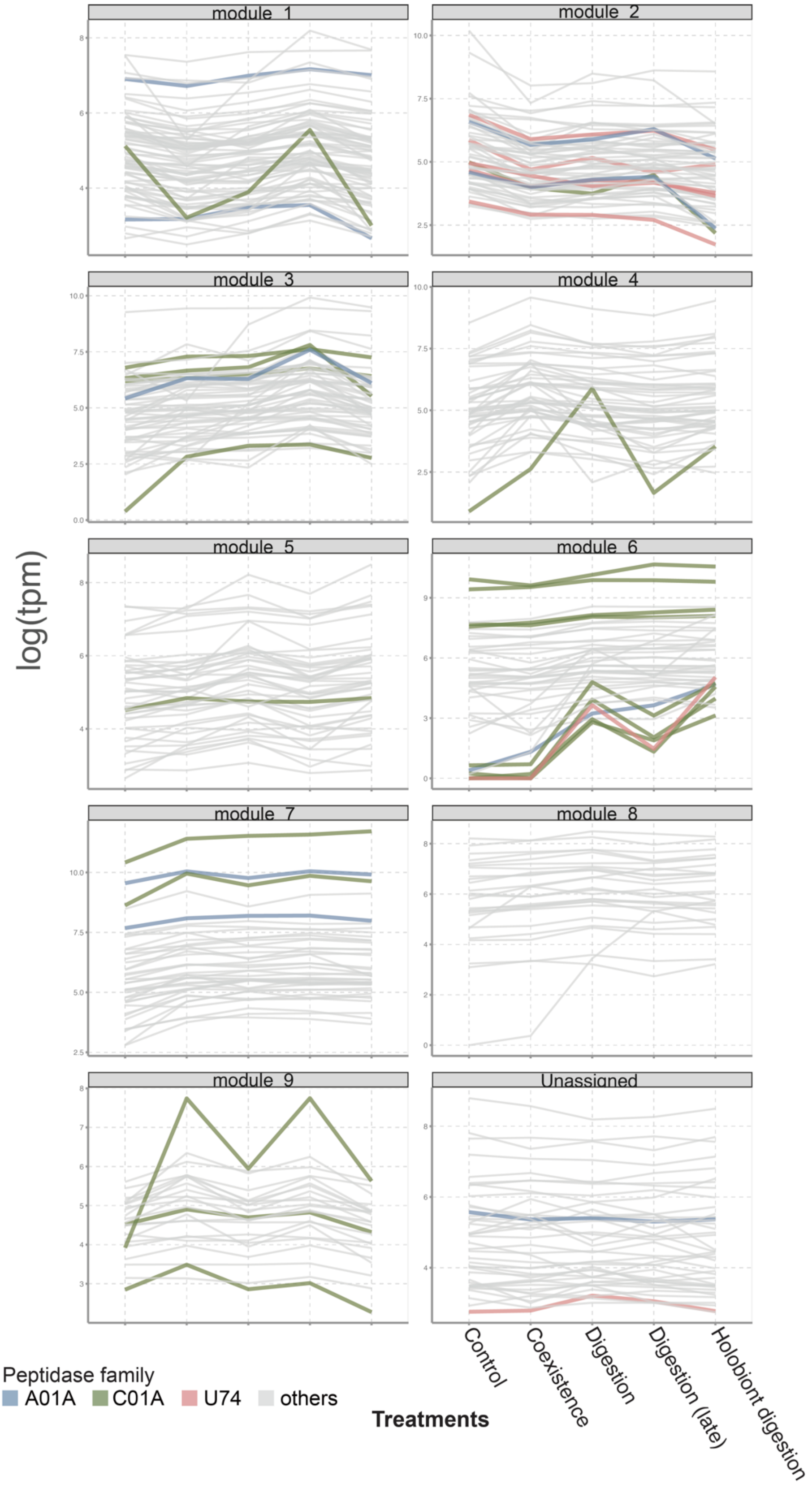

Supplementary Fig. 18. Expression of fungal transporters in different treatments.

Star denote significant upregulation in the holobiont digestion phase compared to either digestion or coexistence process.

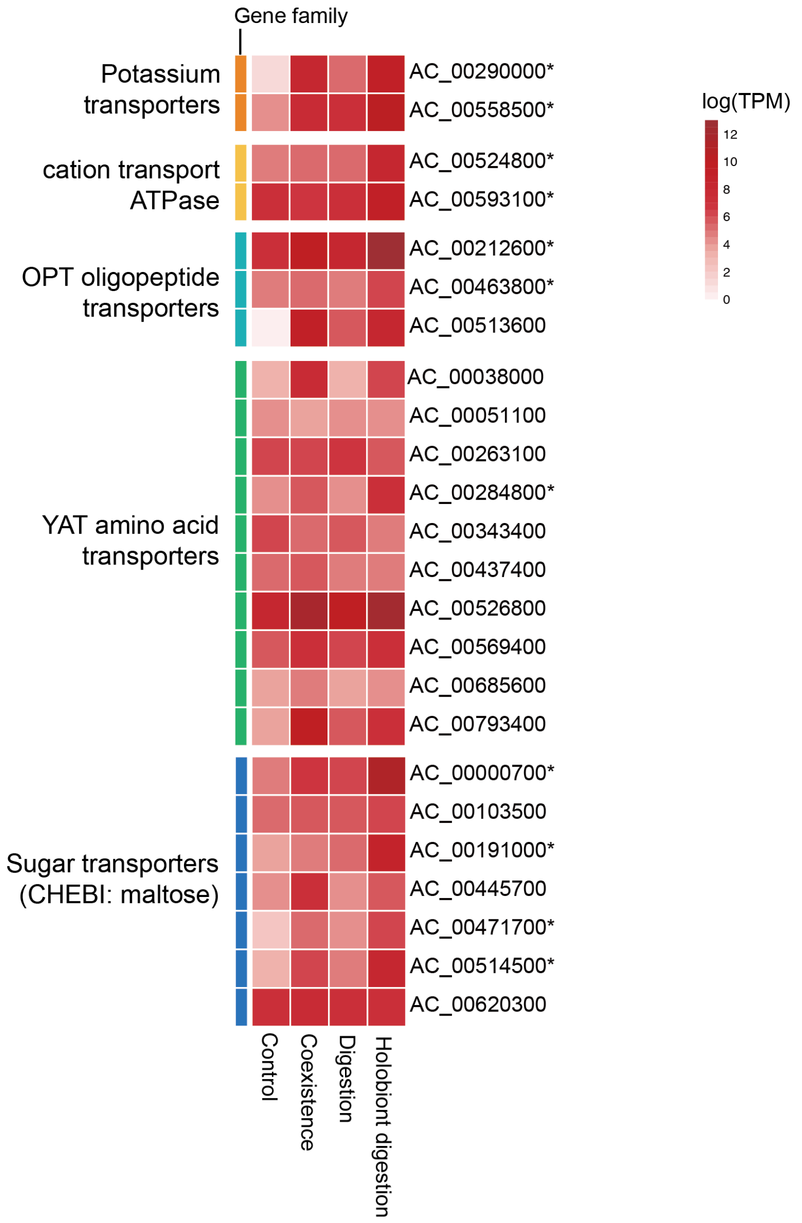

Supplementary Fig. 19. Expression of genes involved in the JA signalling pathway during different phases. Asterisk denote genes that exhibited highest expression in either early or late digestion phase

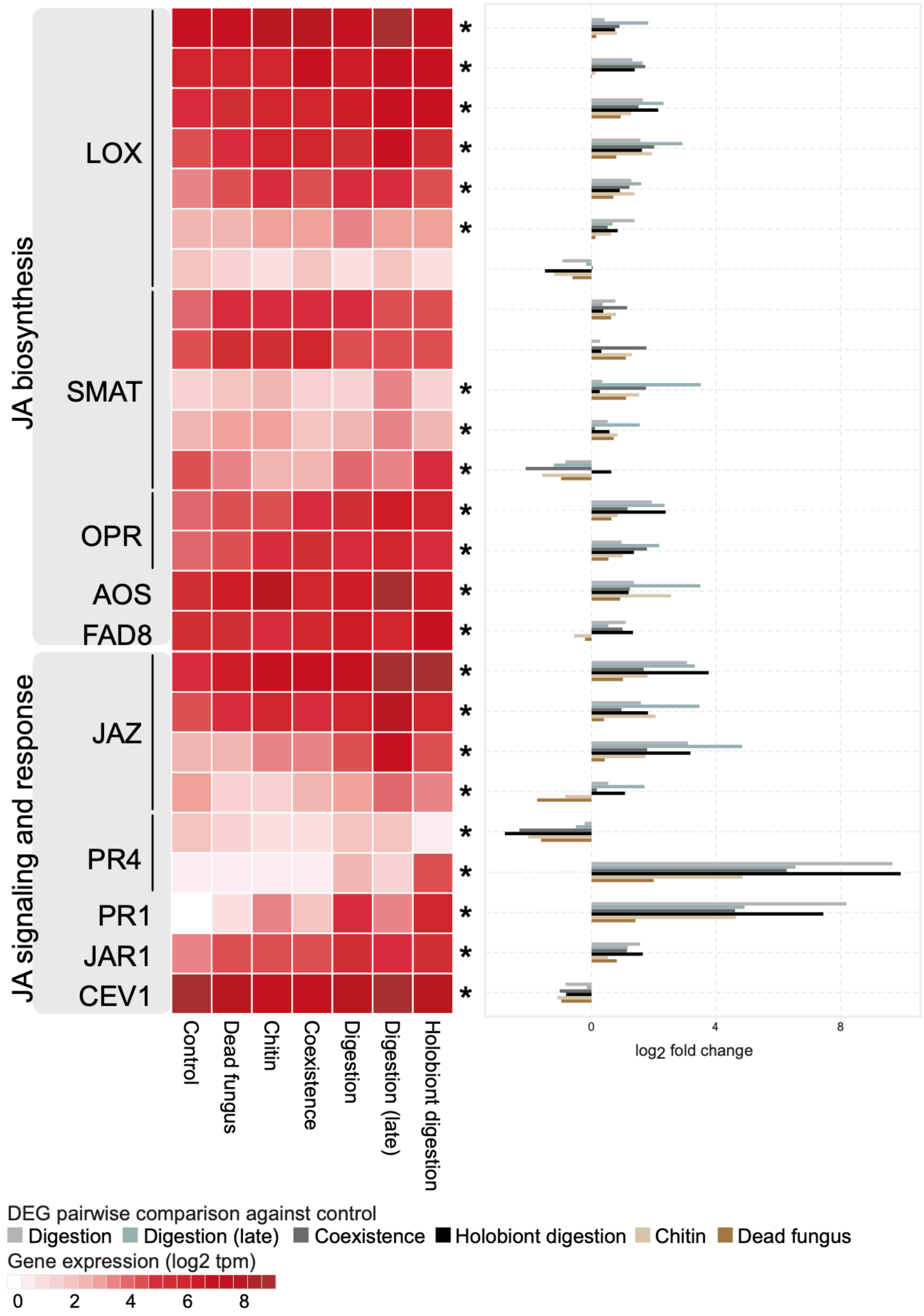

Supplementary Fig. 20. Accumulation of Jasmonoyl-L-isoleucine (JA-Ile) levels in *D. spatulata* following treatments with added chitin, BSA protein, ant insect prey, and inoculated with *A. crateriforme* or the pathogenic *Ph. herbarum*. (Wilcoxon rank sum test; two sided, * P<0.05. ** P<0.01, *** P<0.001; P value control-chitin 0.016, control-ant: 0.008, control- *A. crateriforme:* 0.008, control- *Ph. herbarum*: 0.032, ant-chitin: 0.008, ant- *Ph. herbarum*: 0.008). Each experiment consists of single leaf from five separate plants. Data are presented as mean values +/- SD.

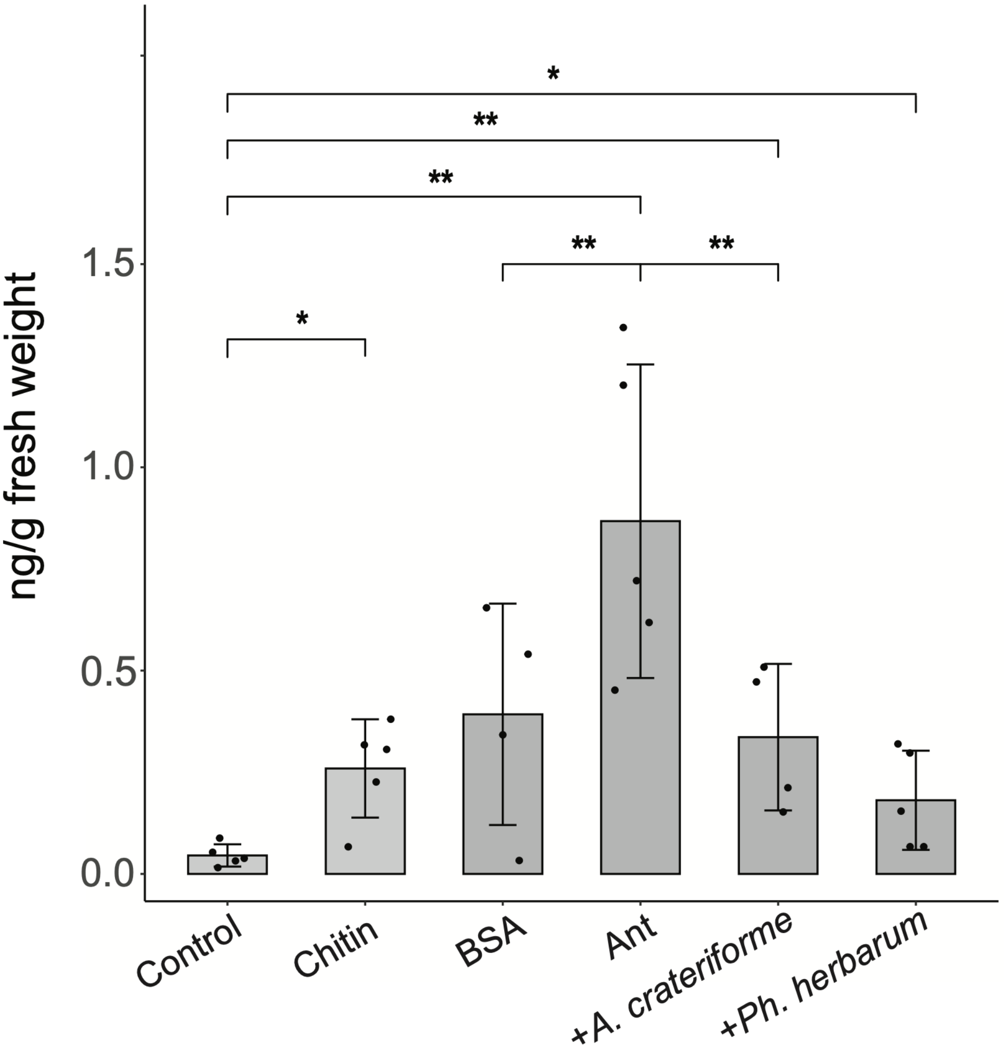

Supplementary Fig. 21. Quantification of jasmonic acid (JA) and salicyclic acid (SA) levels in *D. spatulata* following treatments with added chitin, BSA protein, ant insect prey, and inoculated with *A. crateriforme* or the pathogenic *Ph. herbarum*. (Wilcoxon rank sum test; two sided, * P<0.05. ** P<0.01, *** P<0.001; P value: control- *A crateriforme*: 1.97e-06, control- *A crateriforme* with ant: 0.00028, control- ant: 0.0012, control- BSA: 0.0039, control- chitin: 0.00102, control- *Ph. herbarum:* 0.00029). Each experiment consists of single leaf from ten separate plants. Data are presented as mean values +/- SD.

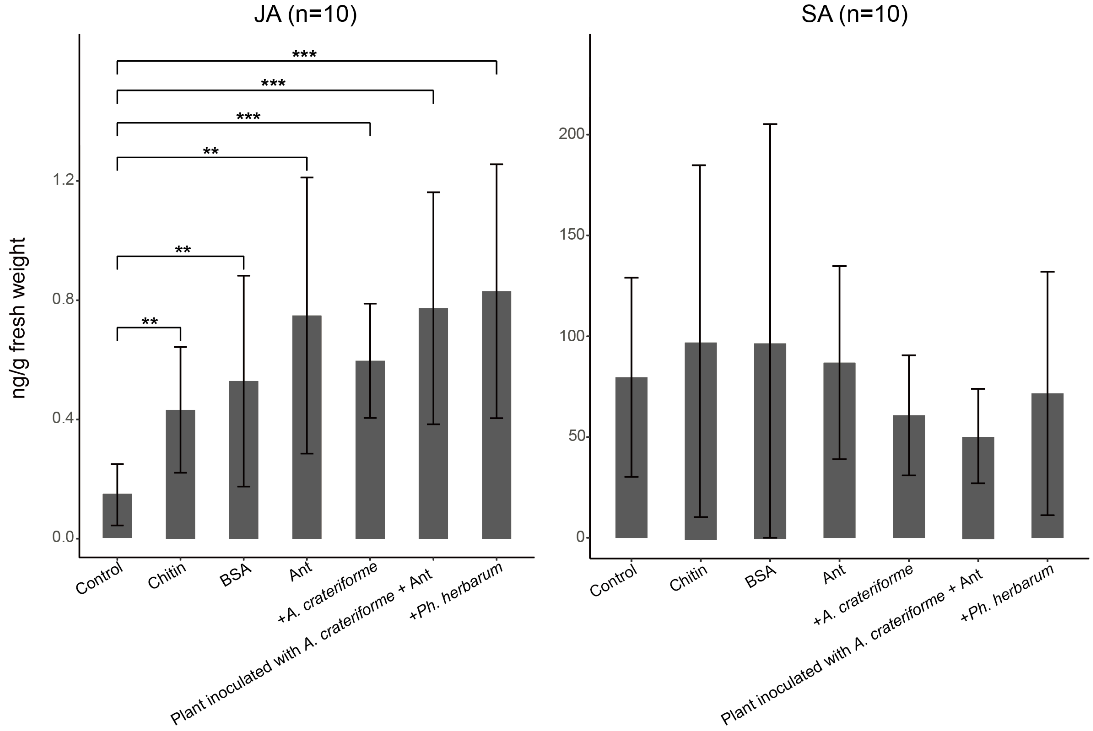

Supplementary Fig. 22. Raw western blot gel image for Fig. 3

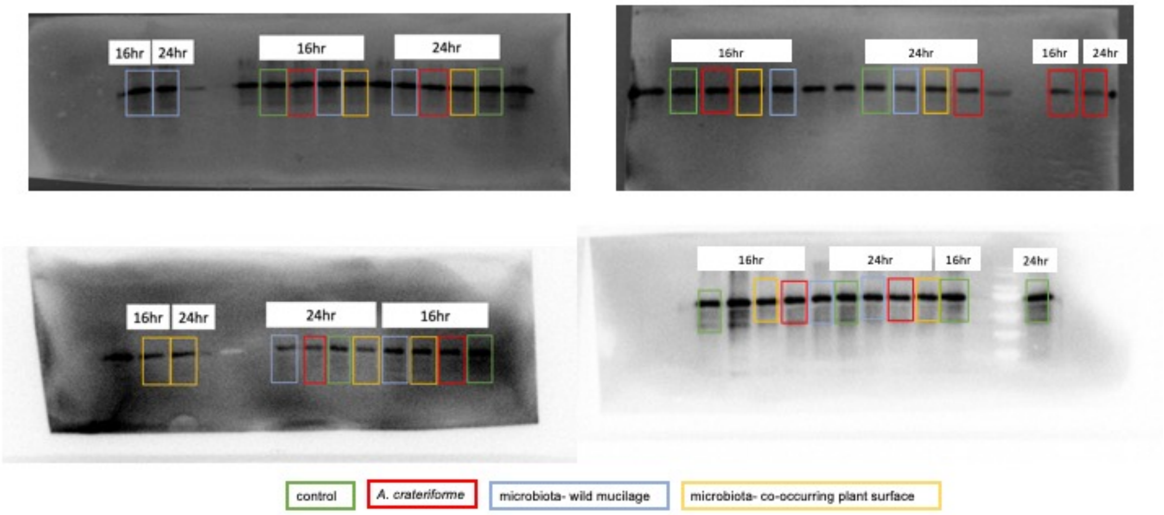

Supplementary Fig. 23. Raw western blot gel image for Extended Fig. 6a

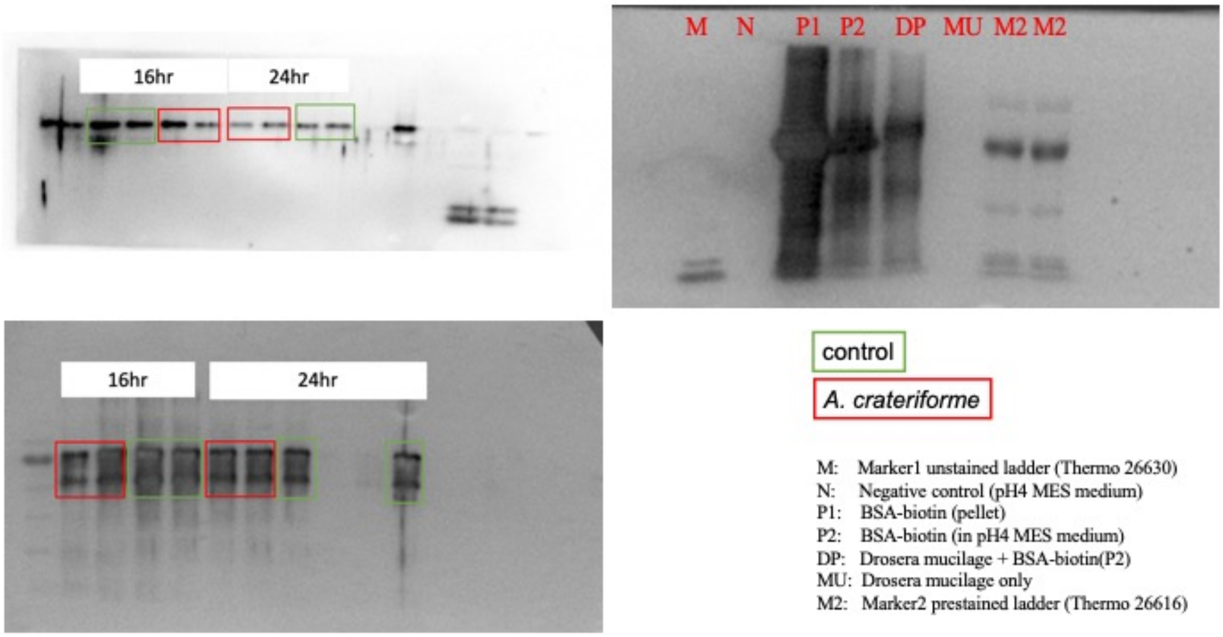

Supplementary Fig. 24. Raw western blot gel image for Extended Fig. 6b

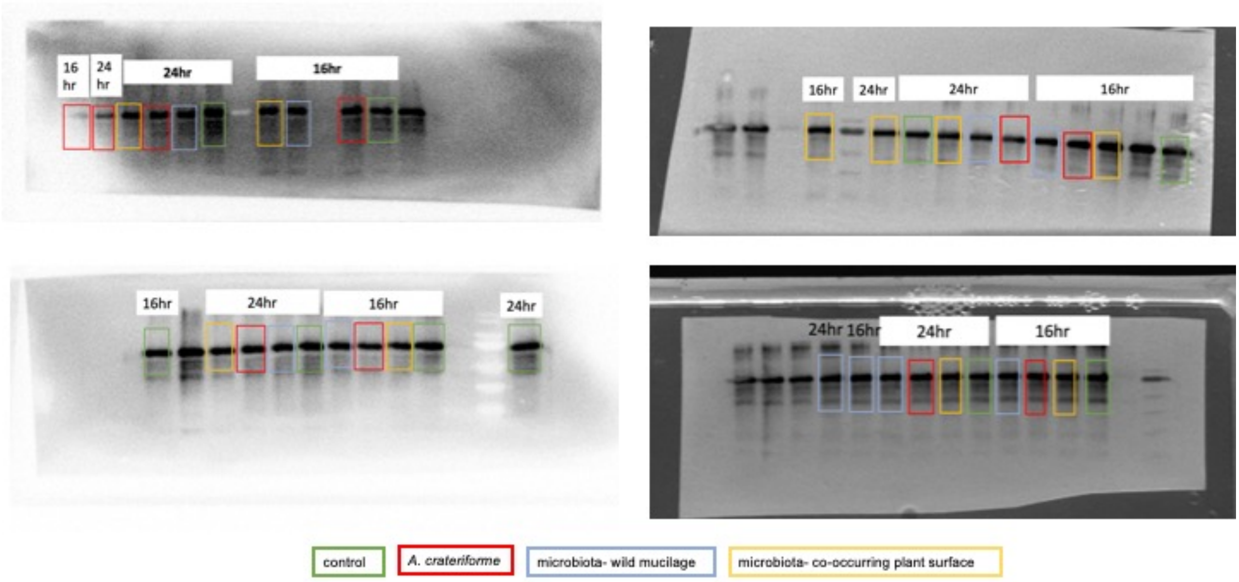

Supplement: Supplementary file 1 — Supplementary Figs. 1–24. [file 41564_2024_1766_MOESM1_ESM.pdf]
