# Supplementary material for: An acidophilic fungus promotes prey digestion in a carnivorous plant
Source: Nat Microbiol. 2024 Aug 1;9(10):2522–37. doi: 10.1038/s41564-024-01766-y (PMC11445062; doi:10.1038/s41564-024-01766-y)
Supplement: Supplementary file 2 — Reporting Summary [file 41564_2024_1766_MOESM2_ESM.pdf]

nature portfolio

Corresponding author(s): Isheng Jason Tsai

Last updated by author(s): Jun 14, 2024

Reporting Summary

Nature Portfolio wishes to improve the reproducibility of the work that we publish. This form provides structure for consistency and transparency in reporting. For further information on Nature Portfolio policies, see our Editorial Policies and the Editorial Policy Checklist.

Statistics

For all statistical analyses, confirm that the following items are present in the figure legend, table legend, main text, or Methods section.

n/a	Confirmed	
☐	☒	The exact sample size (*n*) for each experimental group/condition, given as a discrete number and unit of measurement
☐	☒	A statement on whether measurements were taken from distinct samples or whether the same sample was measured repeatedly
☐	☒	The statistical test(s) used AND whether they are one- or two-sided<br>*Only common tests should be described solely by name; describe more complex techniques in the Methods section.*
☒	☐	A description of all covariates tested
☒	☐	A description of any assumptions or corrections, such as tests of normality and adjustment for multiple comparisons
☐	☒	A full description of the statistical parameters including central tendency (e.g. means) or other basic estimates (e.g. regression coefficient) AND variation (e.g. standard deviation) or associated estimates of uncertainty (e.g. confidence intervals)
☐	☒	For null hypothesis testing, the test statistic (e.g. *F*, *t*, *r*) with confidence intervals, effect sizes, degrees of freedom and *P* value noted<br>*Give P values as exact values whenever suitable.*
☒	☐	For Bayesian analysis, information on the choice of priors and Markov chain Monte Carlo settings
☒	☐	For hierarchical and complex designs, identification of the appropriate level for tests and full reporting of outcomes
☒	☐	Estimates of effect sizes (e.g. Cohen's *d*, Pearson's *r*), indicating how they were calculated

Our web collection on statistics for biologists contains articles on many of the points above.

Software and code

Policy information about availability of computer code

Data collection

32 genomes from representative fungi and plants were downloaded from JGI and NCBI databases
https://mycocosm.jgi.doe.gov/Sepmu1/Sepmu1.home.html
https://mycocosm.jgi.doe.gov/Cerzm1/Cerzm1.home.html
https://www.ncbi.nlm.nih.gov/datasets/genome/GCF_002742065.1/
https://mycocosm.jgi.doe.gov/Mycfi2/Mycfi2.home.html
https://mycocosm.jgi.doe.gov/Clafu1/Clafu1.home.html
https://mycocosm.jgi.doe.gov/Dotse1/Dotse1.home.html
https://mycocosm.jgi.doe.gov/Zasce1/Zasce1.home.html
https://mycocosm.jgi.doe.gov/Mycgr3/Mycgr3.home.html
https://mycocosm.jgi.doe.gov/Disac1/Disac1.home.html
https://mycocosm.jgi.doe.gov/Pieho1_1/Pieho1_1.home.html
https://mycocosm.jgi.doe.gov/Aciri1_iso/Aciri1_iso.home.html
https://mycocosm.jgi.doe.gov/Ternu1/Ternu1.home.html
https://mycocosm.jgi.doe.gov/Horth1/Horth1.home.html
https://mycocosm.jgi.doe.gov/Horwer1/Horwer1.home.html
https://www.ncbi.nlm.nih.gov/datasets/genome/GCA_005059865.1/
https://www.ncbi.nlm.nih.gov/datasets/genome/GCA_030411845.1/
https://mycocosm.jgi.doe.gov/Bauco1/Bauco1.home.html
https://mycocosm.jgi.doe.gov/Horac1/Horac1.home.html
https://mycocosm.jgi.doe.gov/Polci1/Polci1.home.html
https://mycocosm.jgi.doe.gov/Aurpu_var_nam1/Aurpu_var_nam1.home.html

https://mycocosm.jgi.doe.gov/Elsamp1/Elsamp1.home.html
https://www.ncbi.nlm.nih.gov/datasets/genome/GCA_012977835.1/
https://mycocosm.jgi.doe.gov/Myrdu1/Myrdu1.home.html
https://www.ncbi.nlm.nih.gov/datasets/genome/GCF_000146915.1/
SILVA database (v138.1) and the UNITE90 database (v9)
Uniprot Fungi (version October 2019)

Data analysis

Comparative genomics and phylogenomics were using: Orthofinder (ver. 2.5.5), mafft (version 7.741), IQtree (version 2.2.2.6), ASTRAL-III (ver. 5.7.1) and DOLLOP (ver. 3.69.650). Transcriptome analysis were performed using fastp (v0.23.2), STAR (v 2.7.10b), featureCounts (v 2.0.3), DESeq2 (v1.38.3), and topGO (v2.50.0). Genome sequencing, assembly and annotation of A. crateriforme were performed using the canu assembler (ver. 1.9), the flye assembler (ver. 2.5), Racon (four iterations; ver. 1.4.11), Medaka (ver. 0.11.0), Pilon(ver. 1.24) and NOVOPlasty (ver. NOVOPlasty2.7.O.pl). Gene prediction and annotation were performed using open-source tools: STAR (ver. 2.7.7a), Trinity (ver 2.13.2), Trinity (ver 2.13.2; guided approach), Stringtie (ver 2.1.7), Cufflinks (ver2.2.1), Minimap2 (ver 2.1), Portcullis (ver 1.2.3), Augustus (ver 3.4.0), BRAKER2 (ver. 2.1.6), SNAP (ver 2006-07-28), MIKADO (ver 2.3.3), Uniprot Fungi (version October 2019), MAKER2 (ver 3.01.03), Repeatmasker (ver 4.1.2). pfam_scan (ver. 1.6), Pfam database (ver. 36), Diamond (ver. 2.1.6), TransportDB (ver. 2.0), dbCAN (ver. 2.0.11), MEROPS (ver. 12.4), antiSMASH (fungi ver. 7.0.1) and eggNOG (ver. 2.1.12). Amplicon samples were demultiplexed using sabre (v1.0), USEARCH (v11.0.667), phyloseq package (v1.46.0), SILVA database (v138.1) and UNITE database (v.9), rgbif package (v3.7.7).

Analysis and visualisations were conducted under R environment (ver. 4.3.1). Various packages were utilised: topGO79 (ver. 2.52.0), pheatmap (ver. 1.0.12) and ggplot2122 (ver. 3.4.4). The amount of protein remaining and the growth area of A. crateriforme were measured using ImageJ (v 1.53).

For manuscripts utilizing custom algorithms or software that are central to the research but not yet described in published literature, software must be made available to editors and reviewers. We strongly encourage code deposition in a community repository (e.g. GitHub). See the Nature Portfolio guidelines for submitting code & software for further information.

Data

Policy information about availability of data

All manuscripts must include a data availability statement. This statement should provide the following information, where applicable:

- Accession codes, unique identifiers, or web links for publicly available datasets
- A description of any restrictions on data availability
- For clinical datasets or third party data, please ensure that the statement adheres to our policy

All sequences generated from this study were deposited on NCBI under BioProject PRJNA1034788 and PRJNA1095839. The genome and annotation of A. crateriforme is available in NBCI under accession GCA_033807595.1
Accession numbers of individual samples can be found in Supplementary Table 1 and 11.

Research involving human participants, their data, or biological material

Policy information about studies with human participants or human data. See also policy information about sex, gender (identity/presentation), and sexual orientation and race, ethnicity and racism.

Reporting on sex and gender	N/A
Reporting on race, ethnicity, or other socially relevant groupings	N/A
Population characteristics	N/A
Recruitment	N/A
Ethics oversight	N/A

Note that full information on the approval of the study protocol must also be provided in the manuscript.

Field-specific reporting

Please select the one below that is the best fit for your research. If you are not sure, read the appropriate sections before making your selection.

☐ Life sciences ☐ Behavioural & social sciences ☒ Ecological, evolutionary & environmental sciences

For a reference copy of the document with all sections, see nature.com/documents/nr-reporting-summary-flat.pdf

Ecological, evolutionary & environmental sciences study design

All studies must disclose on these points even when the disclosure is negative.

Study description	We collected D. spatulata mucilage samples from five collection sites located in Northern Taiwan. We also collected multiple Drosera species in the UK and USA
Research sample	Drosera spatulata mucilage and co-occurring plants; multiple Drosera tissues
Sampling strategy	*Note the sampling procedure. Describe the statistical methods that were used to predetermine sample size OR if no sample-size calculation was performed, describe how sample sizes were chosen and provide a rationale for why these sample sizes are sufficient.*
Data collection	The fresh leaves of plants and mosses surrounding D. spatulata were also wiped and considered as environmental samples. Each sample is a pooled mucilage from 30 D. spatulata leaves of the same site by using filter paper of size 1 cm x 1.5 cm.
Timing and spatial scale	Sample collecting date is started from June 7th, 2018 to July 28th, 2021. In temporal experiment, we collected sample from June 7th, 2018 to April 23th. we collect samples once a month in Sumei (25.1014697568718N, 121.860991424351E) and Shuangxi (25.066234N, 121.843341E). In spatial experiment, we collect 18 different location (Buyanting: 25.090083N,121.847242E; Houtong: 25.086944N, 121.827778E; Keelung1: 25.159125N, 121.704949E; Keelung2: 25.155061N, 121.710643E, Keelung3: 25.158397N, 121.709447E; Nangang: 25.047277N, 121.639736E; Pingxi1: 25.06611N, 121.71027E; Pingxi2: 25.061111N, 121.78554E; Pingxi3: 25.07416N, 121.79306E; Pingxi4: 24.992262N, 121.663487E; Shiding1: 24.981645N, 121.614058E; Shiding2: 24.97312N, 121.615112E; Shiding3: 24.954655N, 121.624628E; Shuangxi2: 24.970089N, 121.825341E; Shuangxi3: 24.937508N ,121.837337E; Yilan1: 24.85931N, 121.77751E; Yilan2: 24.84593N, 121.77647E) from July 6th, 2021 to July 28th 2021. To compare D. spatulata mucilage and the adjacent environment, we collect samples in Shuangxi(25.066234N, 121.843341E), Shumei(25.1014697568718N, 121.860991424351E) and Yangmingshan(25.181007N, 121.555332E; 25.1769170520255N, 121.558009143632E; 25.1800240781841N, 121.549426075361E) during July, 2019.
Data exclusions	Two ITS rRNA gene amplicon samples were excluded from our analyses due to low read numbers.
Reproducibility	N/A for community experiment
Randomization	D. spatulata mucilage samples and the adjacent environmental samples were randomly collected into the experimental groups
Blinding	Blind testing was not directly relevant to the experiment.

Did the study involve field work? ☒ Yes ☐ No

Field work, collection and transport

Field conditions	The research was studied between June, 2018 to April ,2019.
Location	Drosera spatulata were located in Sumei (25.1014697568718N, 121.860991424351E; 24.970089N, 121.825341E; 24.937508N ,121.837337E), Shuangxi (25.066234N, 121.843341E), Buyanting (25.090083N,121.847242E), Houtong (25.086944N, 121.827778E), Keelung (25.159125N, 121.704949E; 25.155061N, 121.710643E; 25.158397N, 121.709447E), Nangang (25.047277N, 121.639736E), Pingxi (25.06611N, 121.71027E; 25.061111N, 121.78554E; 25.07416N, 121.79306E; 24.992262N, 121.663487E), Shiding (24.981645N, 121.614058E; 24.97312N, 121.615112E; 24.954655N, 121.624628E) Yilan (24.85931N, 121.77751E; 24.84593N, 121.77647E) and Yangmingshan(25.181007N, 121.555332E; 25.1769170520255N, 121.558009143632E; 25.1800240781841N, 121.549426075361E).
Access & import/export	The fresh leaves of plants and mosses surrounding D. spatulata were also wiped and considered as environmental samples. Each sample is a pooled mucilage from 30 D. spatulata leaves of the same site by using filter paper of size 1 cm x 1.5 cm.
Disturbance	The disturbance of the environment by sampling was minimal.

Reporting for specific materials, systems and methods

We require information from authors about some types of materials, experimental systems and methods used in many studies. Here, indicate whether each material, system or method listed is relevant to your study. If you are not sure if a list item applies to your research, read the appropriate section before selecting a response.

Materials & experimental systems

n/a	Involved in the study
☐	☒ Antibodies
☒	☐ Eukaryotic cell lines
☒	☐ Palaeontology and archaeology
☒	☐ Animals and other organisms
☒	☐ Clinical data
☒	☐ Dual use research of concern
☐	☒ Plants

Methods

n/a	Involved in the study
☒	☐ ChIP-seq
☒	☐ Flow cytometry
☒	☐ MRI-based neuroimaging

Antibodies

Antibodies used: Horseradish peroxidase (HRP)-conjugated streptavidin (Cat No. SA00001-0) Dilution rate: (1:5000).

Validation: Streptavidin for biotin. Applications for ELISA, WB, Dot blot.

Dual use research of concern

Policy information about dual use research of concern

Hazards

Could the accidental, deliberate or reckless misuse of agents or technologies generated in the work, or the application of information presented in the manuscript, pose a threat to:

No	Yes
☒	☐ Public health
☒	☐ National security
☒	☐ Crops and/or livestock
☒	☐ Ecosystems
☒	☐ Any other significant area

Experiments of concern

Does the work involve any of these experiments of concern:

No	Yes
☒	☐ Demonstrate how to render a vaccine ineffective
☒	☐ Confer resistance to therapeutically useful antibiotics or antiviral agents
☒	☐ Enhance the virulence of a pathogen or render a nonpathogen virulent
☒	☐ Increase transmissibility of a pathogen
☒	☐ Alter the host range of a pathogen
☒	☐ Enable evasion of diagnostic/detection modalities
☒	☐ Enable the weaponization of a biological agent or toxin
☒	☐ Any other potentially harmful combination of experiments and agents

Plants

Seed stocks	drosera spatulata was collected in Yangmingshan(25.1769170520255N, 121.558009143632E) in July, 2018. seeds were sterilised by 70% ethanol and 3% (w/v) calcium hypochlorite ($CaCl2O2$). Surface sterilised seeds were pregerminated on 0.5% water agar at 20°C for 6-8 weeks. Shoots were then transferred to 1/2 MS agar with pH adjusted to 5.7.
Novel plant genotypes	No novel plant genotypes in this manuscript
Authentication	No authentication in this manuscript
